# Retraction: Induction of Interferon-Stimulated Genes by IRF3 Promotes Replication of *Toxoplasma gondii*

**DOI:** 10.1371/journal.ppat.1012264

**Published:** 2024-05-31

**Authors:** The PLOS Pathogens Editors

After this article [1] was published, concerns were raised about results presented in Figs 1 and 3–6.

Specifically:

- The bottom TgSAG1 panel in Fig 1C appears similar to the middle TgSAG1 panel in Fig 4B.
- The bottom left Actin panel in Fig 1D appears similar to the IRF3 panel in Fig 4C.
- The top right MEF TBK1 panel in Fig 4A appears similar to the 293-GS Actin panel in Fig 6B.
- In several western blot panels in Figs 3B, 3C, 4, 5A and 5C, when levels are adjusted to visualize the background, there are areas within the blot images that appear discontinuous with adjacent regions. For example, in several panels the areas around bands in lanes 2–4 appear discontinuous with neighboring regions and there is no discernible signal in lane 1.

In response to the concerns about similar panels in Figs 1, 4 and 6, the first author stated that the IRF3 panel in Fig 4C, the top right TBK1 panel in Fig 4A and the middle TgSAG1 panel in Fig 4B are incorrect. The first author provided repeat blot images for most experiments reported in Figs 3–5. A member of the *PLOS Pathogens* Editorial Board advised that the repeat data did not support the article’s claims based on Figs 4B and 5C.

The first author provided raw uncropped images for the repeat experiments and stated that the raw data underlying the published figures are no longer available. In the absence of the original uncropped and unadjusted image data underlying the published blot figures, the concerns described in the fourth bullet point, above, cannot be resolved.

The unresolved concerns discussed above call into question the reliability and validity of the reported results and conclusions. Therefore, the *PLOS Pathogens* Editors retract this article.

TM did not agree with the retraction and stands by the article’s findings. The other authors either did not respond directly or could not be reached.
